# Rapid determination of sphingosine 1-phosphate association with carrier molecules by flow-induced dispersion analysis to predict sepsis outcome

**DOI:** 10.1016/j.isci.2024.111168

**Published:** 2024-10-15

**Authors:** Isabelle Seidita, Anke Ziegler, Auron Qalaj, Martin Sebastian Winkler, Axel Nierhaus, Stefan Kluge, Bodo Levkau, Markus H. Gräler

**Affiliations:** 1 Department of Anesthesiology and Intensive Care Medicine, Center for Molecular Biomedicine (CMB), Jena University Hospital, Hans-Knöll-Straße 2, D-07745 Jena, Germany; 2 Department of Anesthesiology, Emergency and Intensive Care Medicine, University of Göttingen, Robert-Koch-Straße 40, 37075 Göttingen, Germany; 3 Department of Intensive Care, Universitätsklinikum Eppendorf, Martinistraße 52, 20246 Hamburg, Germany; 4 Institute of Molecular Medicine III, Heinrich Heine University, Universitätsstraße 1, 40225 Düsseldorf, Germany; 5 Center for Sepsis Control and Care, Jena University Hospital, 07740 Jena, Germany

**Keywords:** clinical measurement in health technology, lipid, biochemical assay

## Abstract

Flow-induced dispersion analysis (FIDA) was used to investigate the association of fluorescein isothiocyanate-labeled signaling lipid sphingosine 1-phosphate (S1P) with its carrier molecules human serum albumin (HSA) and high-density lipoprotein (HDL). Associations were measured in plasma samples of patients after surgery, with sepsis or septic shock. All patients demonstrated a significant shift between the carrier binding: decrease of S1P bound to HSA with a concomitant increase of S1P bound to HDL. The molecular sizes of binding complexes correlated well with the relative amounts of S1P bound to HSA and HDL detected by liquid chromatography-tandem mass spectrometry. Very low complex formation of S1P with HDL was observed in several septic shock patients and correlated with the need for mechanical ventilation and intensive care unit (ICU) mortality. Determination of S1P binding to HSA and HDL by FIDA could therefore be useful in the clinical setting to predict disease progression, severity, and outcome.

## Highlights

• Flow-induced dispersion analysis (FIDA) to quickly monitor S1P carrier in plasma
• Relative decrease of HSA-S1P and increase of HDL-S1P in sepsis
• Confirmation of FIDA results by LC-MS/MS
• Low HDL-S1P in septic shock patients correlates with ICU mortality

## Graphical abstract

**Figure:**
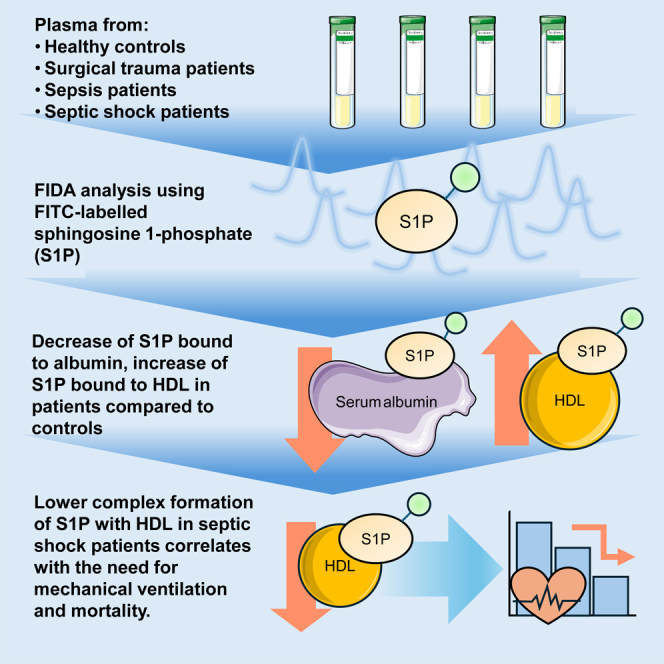

## Introduction

Flow-induced dispersion analysis (FIDA) was developed to investigate changes of the size of molecular complexes induced by biophysical events like binding, protein (un-)folding, degradation, and synthesis.^1^ FIDA determines the diffusion coefficients of molecules and complexes and converts them to hydrodynamic radii (R_h_) as an absolute measure of the molecular size via the Stokes-Einstein equation.^2^ Molecules are detected by emission of a fluorescent signal that can also serve as a quantitative measure. A fluorescent indicator and an analyte containing the potential binding partner are mixed in a glass capillary under constant flow. The molecules in solution will disperse depending on their size and flow speed. The fluorescent indicator will be detected in a flow cell positioned at the end of the capillary. The width of the resulting fluorescent signal is dependent on the molecular size of the fluorescent molecule. In the presence of a binding partner in the analyte solution, the size of the fluorescent molecule will increase due to complex formation and consequently result in a broader signal, which translates into an increased molecular size. This method has already been used to study drug interactions in patient samples,^3^^,^^4^ antibody binding,^5^^,^^6^ and protein stability.^2^^,^^7^

Sphingosine 1-phosphate (S1P) is a signaling lipid molecule present in circulation at high nanomolar concentrations.^8^^,^^9^ It binds to apolipoprotein M (ApoM) as part of high-density lipoprotein (HDL) and to human serum albumin (HSA) in blood, both of which serve as carrier proteins.^8^^,^^10^^,^^11^ Binding of S1P to HDL and HSA is highly dynamic and can change in cardiovascular and inflammatory diseases.^12^^,^^13^^,^^14^^,^^15^ Previous studies suggest that differences in S1P binding to its carrier molecules have functional consequences and diagnostic value.^15^^,^^16^^,^^17^ S1P bound to HDL is considered as the main inducer of endothelial-protective and inflammation-dampening effects that are typically connected with HDL function.^12^^,^^18^ On the other hand, S1P also associates with HSA, and HSA supplementation might have a beneficial effect in septic shock patients. In a large multicenter randomized clinical trial, HSA supplementation had little effect in patients with sepsis or severe sepsis, but a *post hoc* analysis of data from septic shock patients demonstrated significantly lower mortality at 90 days.^19^ A potential role of S1P bound to HSA cannot be excluded and requires further investigation. We have recently shown that S1P binding is shifted from HSA to HDL in patients with surgical trauma, sepsis, and septic shock.^17^ Potential functional consequences of the observed displacement of S1P carrier molecules are currently under investigation. Unfortunately, detection of skewed S1P binding to its carrier molecules in plasma with current techniques like lipoprotein precipitation followed by liquid chromatography-tandem mass spectrometry (LC-MS/MS) is error prone, difficult, and time consuming. We therefore adapted FIDA for the rapid determination of S1P binding to its carrier molecules HDL and HSA in plasma samples obtained from healthy volunteers and from patients suffering from surgical trauma, sepsis, and septic shock.

## Results

### Method development to determine S1P-HDL and S1P-HSA binding complexes

**Figure 1:**
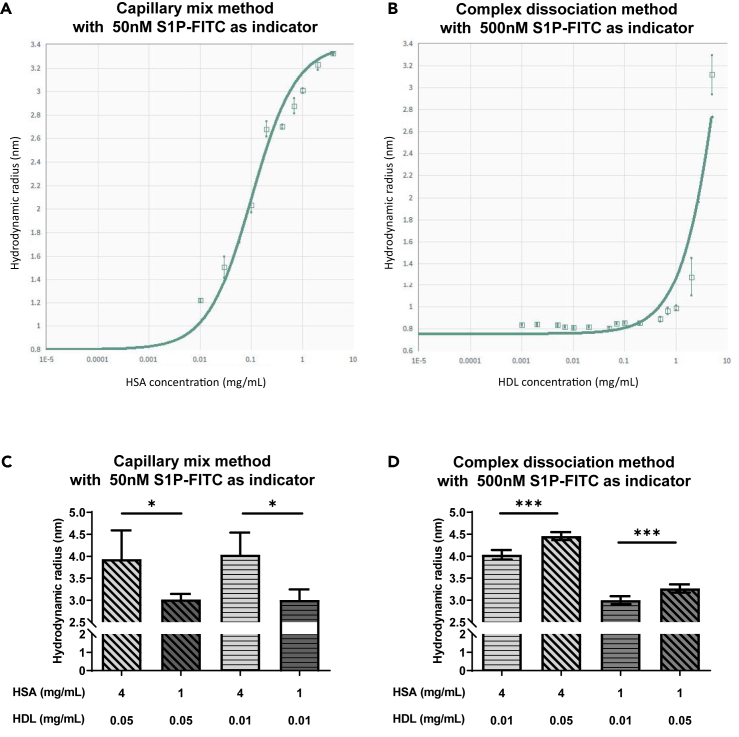
Establishment of FIDA methods for determination of HSA-S1P and HDL-S1P complexes (A) Binding curve of 50 nM S1P-FITC to HSA using the capillary mix method. The dissociation constant K_D_ was determined to be 20 μg/mL. (B) Binding curve of 500 nM S1P-FITC to HDL using the complex dissociation method. The K_D_ was determined to be 1.25 mg/mL protein. (A and B) Shown are means ± SEM, *n* = 3. (C and D) HSA versus HDL competition experiments. (C) 50 nM S1P-FITC was used as indicator, and combinations of HSA and HDL in various concentrations (HSA 1 mg/mL or 4 mg/mL, HDL 0.01 mg/mL or 0.05 mg/mL) were used as analyte. The capillary mix method was used with the capillary heated at 37°C and the sample trays cooled at 4°C. Shown are means ± SD of the R_h_ measured in 4 independent experiments. Differences are significant according to Student’s t test (∗*p* < 0.05). (D) 500 nM S1P-FITC was used as indicator, and combinations of HSA and HDL in various concentrations (HSA 1 mg/mL or 4 mg/mL, HDL 0.01 mg/mL or 0.05 mg/mL) were used as analyte. The complex dissociation method was used with the capillary heated at 37°C and the sample trays cooled at 4°C. Shown are means ± SD of the R_h_ measured in 4 independent experiments. Differences are significant according to Student’s t test (∗∗∗*p* < 0.001).

In order to test the validity of FIDA to investigate binding of S1P to its carrier molecules HDL and HSA in solution, we used 50 nM fluorescein isothiocyanate-labeled S1P (S1P-FITC) as indicator. Therefore, a binding curve was generated using the capillary mix method, which is dependent on the association kinetics of S1P-FITC and HSA. The resulting dissociation constant K_D_ was 20 μg/mL (Figure 1A). For detection of S1P-FITC binding to HDL, we applied the complex dissociation method, which is dependent on the dissociation kinetics of S1P-FITC and HDL. To promote S1P-FITC/HDL complex formation and consequent detection sensitivity, a 10-times higher concentration of 500 nM instead of 50 nM S1P-FITC was used as indicator. The resulting K_D_ was 1.25 mg/mL protein (Figure 1B). Importantly, no specific binding at these concentrations was observed with fluorescein only, demonstrating the specificity of S1P binding to both HDL and HSA. For the capillary mix method, the intraday precision was determined in triplicates to be 1.3%, while the interday precision was 1.6%. Intra- and interday precision of the complex dissociation method was determined to be 0.4%. The high precision is attributable to the first-principle technology of FIDA to determine the diffusion coefficient as an absolute measure, meaning that it does not depend on any external reference point. The polydispersity index (PDI) was below 0.1 for all samples measured with both methods. In FIDA, a PDI value below 0.5 is considered as a monodisperse solution consisting of homogenous complexes, in this case S1P-FITC/HSA for the capillary mix method and S1P-FITC/HDL for the complex dissociation method.^20^ The signal-to-noise (S/N) ratios of the capillary mix method and the complex dissociation method were all above 100 and above 1,000, respectively. S/N ratios above 30 are recommended for FIDA to obtain interpretable results according to the technical specifications of the instrument.

### Discrimination between S1P-HDL and S1P-HSA binding complexes

We next tested the possibility to discriminate complexes of S1P-FITC binding to HDL and HSA in solutions containing different concentrations of the respective analytes. Four mg/mL HSA was used as a physiological concentration and 1 mg/mL as a pathologically low concentration found in 10% plasma. Similarly, 0.05 and 0.01 mg/mL HDL protein were used and mixed with HSA. The capillary mix method with 50 nM S1P-FITC as indicator resulted in a significant drop of the R_h_ with low HSA concentration regardless of the added HDL concentration (Figure 1C). In contrast, the complex dissociation method with 500 nM S1P-FITC as indicator demonstrated a significant increase of the R_h_ with increasing HDL concentration, although lower HSA concentrations still reduced the R_h_ (Figure 1D). Thus, the capillary mix method demonstrated concentration-dependent complex formation of S1P-FITC with HSA independently of the HDL concentration, while the R_h_ determined with the complex dissociation method was dependent on both HSA and HDL concentrations. A potential reason for these different results could be that the association of S1P-FITC with HDL is much slower than that of S1P-FITC with HSA. Consequently, the time provided for complex association in the capillary mix method might not be sufficient to get S1P-FITC/HDL complexes until measurement. On the other hand, samples were pre-equilibrated in the complex dissociation method, so that sufficient time was provided for the assembly of S1P-FITC/HDL complexes.

### Evaluation of FIDA methods with serum and plasma samples

**Figure 2:**
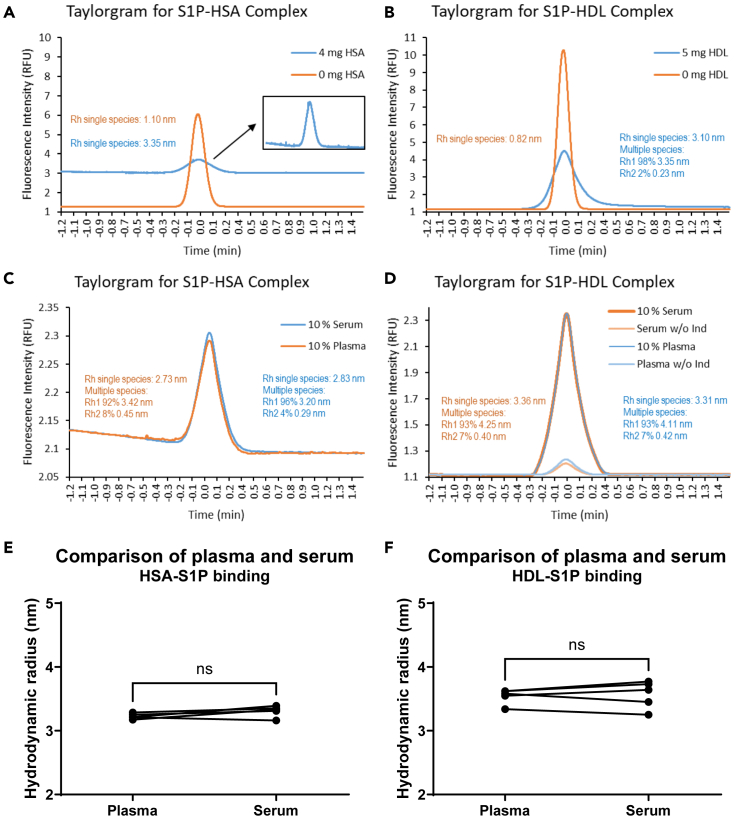
Taylorgrams of FIDA methods and comparison of plasma and serum as analyte (A) Taylorgrams of 4 mg/mL HSA as analyte and 50 nM S1P-FITC as indicator in the capillary mix method. (B) Taylorgrams of 5 mg/mL HDL as analyte and 500 nM S1P-FITC as indicator in the complex dissociation method. (C) Taylorgrams of 10% serum and 10% plasma in the capillary mix method. (D) Taylorgrams of 10% serum and 10% plasma in the complex dissociation method. Samples without addition of the indicator (w/o Ind.) are shown to demonstrate the background signal derived by HSA present in 10% plasma or serum. (E) Comparison of the means of serum and plasma samples derived from five different patients measured in triplicates with the capillary mix method. (F) Comparison of the means of serum and plasma samples derived from five different patients measured in triplicates with the complex dissociation method (ns, not significant).

We proceeded with the evaluation to use human plasma or serum samples in order to determine the relative association of S1P-FITC with HSA or HDL in these biological fluids. Taylorgrams derived from the capillary mix method shown in Figure 1A revealed an increasing background fluorescence derived from HSA with increasing HSA concentrations. An HSA concentration of 4 mg/mL resulted in still acceptable S/N ratios greater than 100 despite the significantly increased background signal compared to pure S1P-FITC (Figure 2A). Taylorgrams of S1P-FITC/HDL complexes from the complex dissociation method shown in Figure 1B revealed normal background up to the highest concentration of 5 mg/mL with S/N ratios greater than 1,000 (Figure 2B). Due to the higher S1P-FITC concentration of 500 nM used in the complex dissociation method, multiple-species analysis revealed a low amount of 2% of free indicator even at the highest HDL concentration of 5 mg/mL, while complex formation of S1P-FITC/HSA with 4 mg/mL HSA and 50 nM S1P-FITC was monodisperse (Figures 2A and 2B). Application of the capillary mix method with 10% human plasma and serum revealed similar Taylorgrams with S/N ratios greater than 100 (Figure 2C). The observed slight baseline drift could be corrected by the analysis software. The concentration of 10% serum and plasma was chosen due to a concentration of HSA close to 4 mg/mL, which proved to produce a sufficiently low background for analysis (Figure 2A). Since the amount of HDL in plasma and serum is about 100-times lower than the amount of HSA (0.5 mg/mL protein content of HDL compared to 35–50 mg/mL HSA), it was important to measure plasma and serum at the highest possible concentration in order to maintain HDL-dependent signals with the complex dissociation method. Multiple species analysis indicated a low amount of 4%–8% of free indicator S1P-FITC, which was important to stay below saturating conditions that would otherwise prevent the discrimination of different samples regarding the ability to form S1P-FITC/HSA complexes. This was similar with the complex dissociation method, which revealed 7% free indicator S1P-FITC at the higher concentration of 500 nM (Figure 2D). To overcome the demonstrated autofluorescence of HSA in the complex dissociation method, a 10-times higher concentration of the indicator S1P-FITC was used (Figure 2D). Again, plasma and serum samples provided similar Taylorgrams with S/N ratios greater than 1,000. Due to the fact that the discrimination of multiple species was not precise enough to provide differences in HDL/HSA mixtures, all samples were subsequently analyzed by the single-species method to obtain results shown in Figures 1C and 1D. The comparison of the obtained R_h_ from five plasma and serum samples measured in triplicates did not provide any significant differences for the capillary mix method (Figure 2E) and the complex dissociation method (Figure 2F), so we decided to continue with the analysis of plasma samples.

### S1P-HDL and S1P-HSA complex formation in patient samples

**Figure 3:**
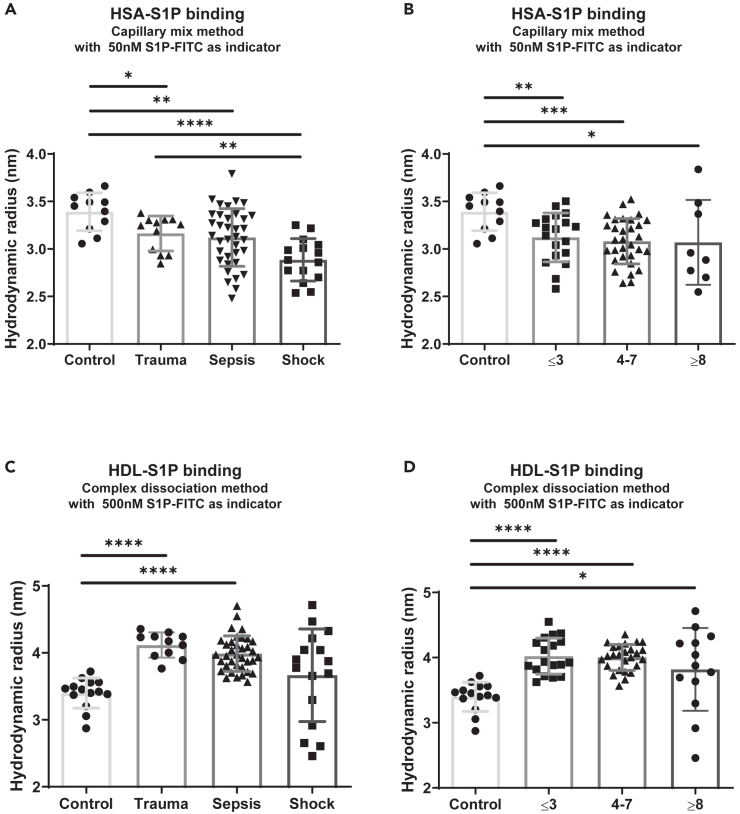
Determination of HSA-S1P and HDL-S1P complex formation in plasma of trauma, sepsis, and septic shock patients compared to healthy controls (A) The R_h_ of HSA-S1P complexes were determined with the capillary mix method using 10% plasma as analyte and 50 nM S1P-FITC as indicator. Reduced amounts of HSA-S1P complexes in trauma, sepsis, and septic shock patients resulted in decreased R_h_ compared to healthy controls. *n* = 11 (control), 11 (trauma), 36 (sepsis), 15 (septic shock). (B) This was also the case after reclassification of the patients according to their SOFA score. *n* = 11 (control), 19 (≤3), 31 (4–7), 8 (≥8). (C) The R_h_ of HDL-S1P complexes were determined with the complex dissociation method using 10% plasma as analyte and 500 nM S1P-FITC as indicator. Increased amounts of HDL-S1P complexes in trauma, sepsis, and septic shock patients resulted in larger R_h_ compared to healthy controls. *n* = 14 (control), 11 (trauma), 36 (sepsis), 16 (septic shock). (D) This was also the case after reclassification of the patients according to their SOFA score. *n* = 14 (control), 18 (≤3), 27 (4–7), 13 (≥8). (A–D) Groups were compared using a non-parametric Mann-Whitney U test (∗*p* < 0.05; ∗∗*p* < 0.01; ∗∗∗*p* < 0.001; ∗∗∗∗*p* < 0.0001). Shown are means ± SD.

**Figure 4:**
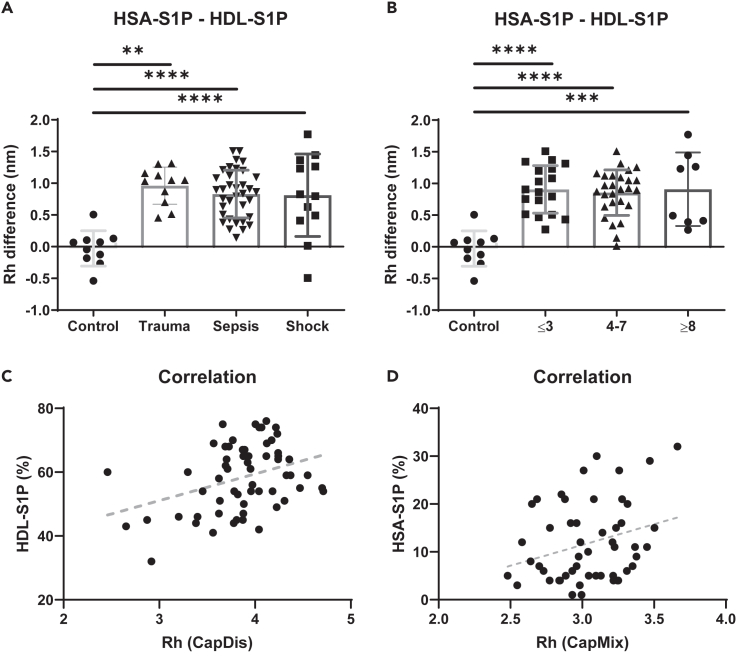
Magnitude of shifted S1P binding from HSA to HDL and correlation with endogenous plasma S1P bound to HSA and HDL as determined by LC-MS/MS (A) The difference in the R_h_ of HSA-S1P complexes subtracted from that of HDL-S1P complexes reveals relative increases of HDL-S1P complexes over HSA-S1P complexes in trauma, sepsis, and septic shock patients compared to healthy controls. *n* = 10 (control), 11 (trauma), 36 (sepsis), 12 (septic shock). (B) This was also the case after reclassification of the patients according to their SOFA score. *n* = 10 (control), 18 (≤3), 27 (4–7), 8 (≥8). (A and B) Shown are means ± SD. (C) Correlation of the R_h_ of HDL-S1P complexes with the relative amount of endogenous S1P bound to HDL. The R_h_ of HDL-S1P complexes positively correlate with the relative amount of endogenous S1P bound to HDL as determined by LC-MS/MS (Spearman r = 0,2838, ∗*p* < 0.05, *n* = 60). (D) Correlation of the R_h_ of HSA-S1P complexes with the relative amount of endogenous S1P bound to HSA. The R_h_ of HSA-S1P complexes positively correlate with the relative amount of endogenous S1P bound to HSA as determined by LC-MS/MS (Spearman r = 0,3190, ∗*p* < 0.05, *n* = 53).

In order to explore the possibility to discriminate the association of S1P with HSA and HDL in plasma samples from patients, we re-analyzed plasma samples from a patient cohort that was previously investigated regarding S1P concentrations in serum and the amount of HDL-S1P and HSA-S1P in plasma.^17^^,^^21^ The patient cohort consisted of 14 healthy volunteers, 11 patients after surgery, 36 sepsis patients, and 16 patients with septic shock (Table 1). Surgical trauma patients were included in this study because they had no infection compared to systemically infected sepsis patients. But they developed a systemic inflammatory response syndrome similar to sepsis patients and were characterized by a low sequential organ failure assessment (SOFA) score (Table 1). We used 10% plasma as analyte and determined the complex sizes formed with 50 and 500 nM S1P-FITC as indicator using the established capillary mix and complex dissociation methods, respectively. The determination of S1P-FITC/HSA complexes using the capillary mix method demonstrated significantly reduced binding of the indicator S1P-FITC to HSA in 10% plasma of patients with surgical trauma, sepsis, and septic shock compared to healthy controls. A significant reduction was also found in septic shock patients compared to surgical trauma patients (Figure 3A). Reduced binding of S1P-FITC was observed in all patients regardless of the severity of disease, measured by the SOFA score (Figure 3B). Importantly, S1P-FITC association with HDL concomitantly increased in 10% plasma of patients with surgical trauma and sepsis, while the septic shock group demonstrated very heterogeneous complex sizes (Figure 3C). Again, all patients demonstrated significantly increased S1P-FITC association with HDL independent from their actual SOFA score (Figure 3D). To discriminate S1P-FITC binding to HDL from HSA, we subtracted the R_h_ determined by the complex dissociation method, which was specific for HDL association, from the R_h_ determined by the capillary mix method, which was specific for HSA association of S1P-FITC. The reasoning behind this was the fact that the R_h_ was similarly affected in both methods by HSA, while HDL influenced only the R_h_ in the complex dissociation method. The resulting data clearly show the shift of S1P-FITC binding from HSA in healthy controls to HDL in patients with surgical trauma, sepsis, and septic shock (Figure 4A). Increased binding of S1P-FITC with HDL and concomitantly reduced association with HSA was again independent from the SOFA score (Figure 4B). The observed reallocation confirmed LC-MS/MS data that demonstrated a shift of endogenous S1P in all patients. In addition, the R_h_ determined by the capillary mix method positively correlated with the relative amount of endogenous S1P bound to HSA (Figure 4C), and the R_h_ determined by the complex dissociation method positively correlated with the relative amount of endogenous S1P bound to HDL (Figure 4D). FIDA results were therefore in line with LC-MS data.

**Table 1:** Characteristics of the study cohort

Clinical parameter	Control	Trauma	Sepsis	Septic shock	*p* value^a^
*n*	14	11	36	16	N/A
Age (years)	31 (27–48)	63 (53–71)	60 (48–68)	67 (60–75)	0.0014
Male (%)	43	42	54	50	ns
SOFA score	N/A	4 (3–5)	4 (2–6)	8 (7–11)	<0.0001
Leukocytes/nL	6.3 (6.0–7.8)	13.4 (11.7–15.3)	12.8 (11.0–16.4)	15.4 (11.7–22.2)	0.0086
Neutrophils/nL	3.5 (2.9–5.5)	10.3 (8.9–12.5)	11.4 (7.0–14.4)	13.5 (9.5–20.6)	0.0029
Lymphocytes/nL	2.1 (1.6–2.4)	1.0 (0.8–1.5)	0.9 (0.5–1.5)	0.8 (0.3–1.1)	0.0055
PCT (μg/L)	N/A	0.3 (0.1–1.5)	0.5 (0.2–1.9)	3.1 (1.0–17.5)	0.0078
Lactate (mM)	N/A	1.0 (0.8–1.7)	1.3 (1.0–1.9)	1.7 (1.3–2.6)	0.0374
Balance (mL)	N/A	404 (-94-1274)	660 (136–1697)	2582 (2085–3522)	0.0010

SOFA, sequential organ failure assessment score; PCT, procalcitonin; N/A, not applicable; ns, not significant. Data are presented as median (interquartile range).

a *p* value for trend between patient groups using a non-parametric ANOVA Kruskal-Wallis test.

### Evaluation of FIDA results and correlations with clinical data

**Figure 5:**
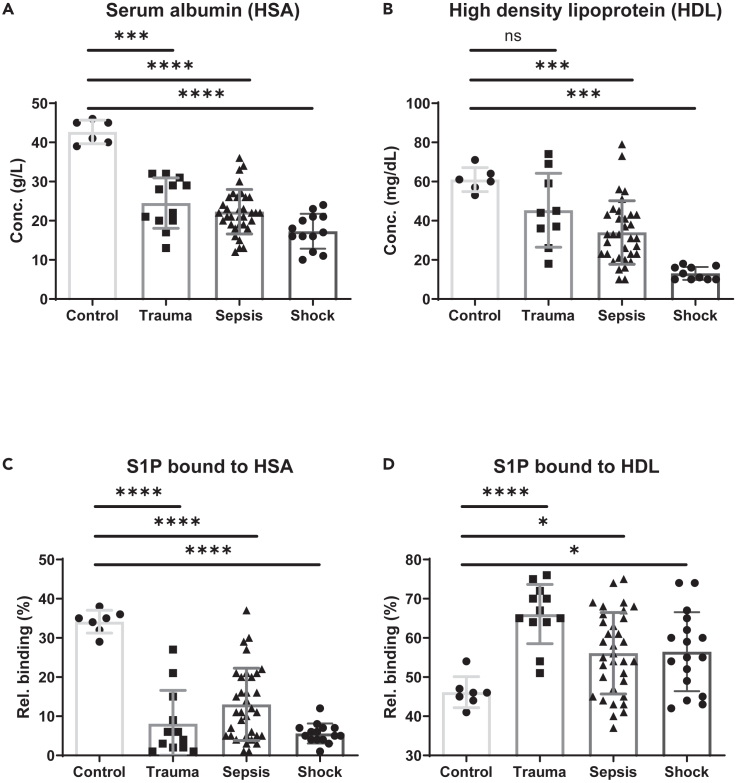
Concentrations of HSA, HDL, and S1P bound to HSA and HDL determined by LC-MS/MS (A and B) Concentration of (A) HSA and (B) HDL in plasma samples of healthy controls and patients with surgical trauma, sepsis, and septic shock. (C and D) Determination of S1P bound to (C) HSA and (D) HDL determined by LC-MS/MS after lipoprotein precipitation. Groups were compared using the non-parametric Mann-Whitney U test (∗*p* < 0.05, ∗∗∗*p* < 0.001, ∗∗∗∗*p* < 0.0001, ns, not significant). Shown are means ± SD, *n* = 7 (control), 11 (trauma), 34 (sepsis), 13 (septic shock).

**Figure 6:**
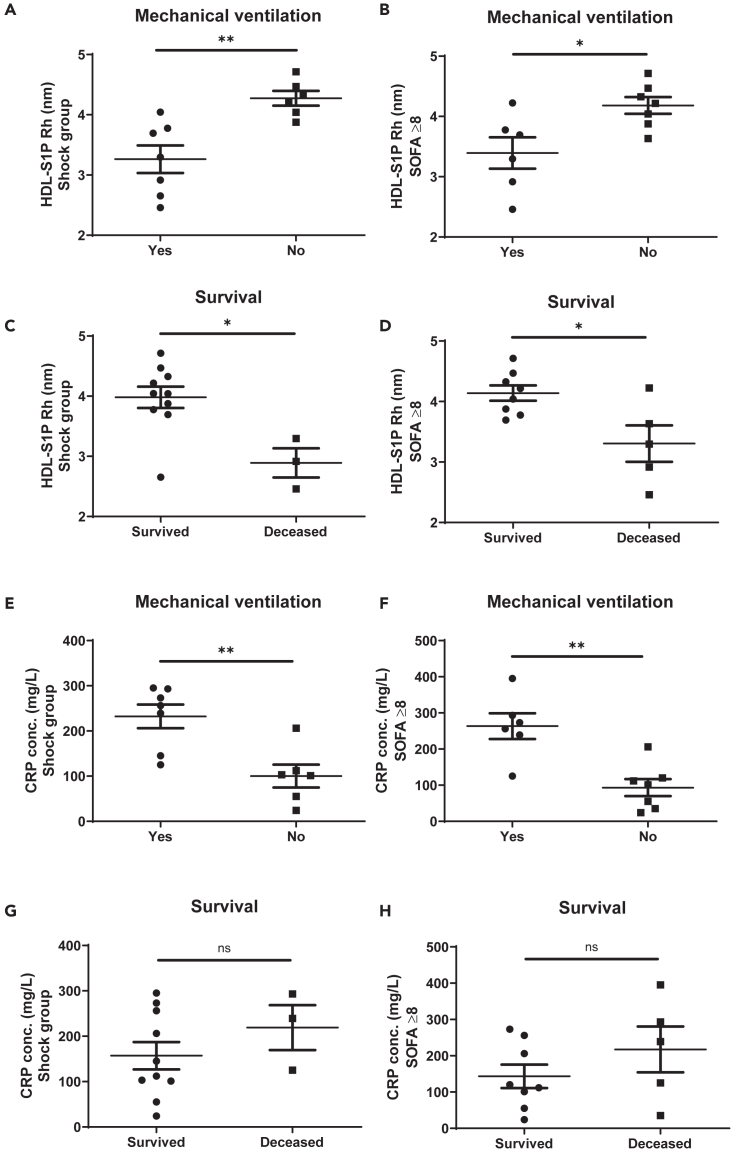
Correlation of the R_h_ of HDL-S1P complexes and the C-reactive protein with the need of mechanical ventilation and survival in the septic shock group and in the reclassified patient group with high SOFA score (A and B) Negative correlation of the R_h_ of HDL-S1P complexes in 10% plasma of (A) septic shock patients and of (B) reclassified patients with high SOFA score with the need for mechanical ventilation. (C and D) Positive correlation of the R_h_ of HDL-S1P complexes in 10% plasma of (C) septic shock patients and of (D) reclassified patients with high SOFA score with survival. (E and F) Positive correlation of the CRP concentration in plasma of (E) septic shock patients and of of (F) reclassified patients with high SOFA score with the need for mechanical ventilation. (G and H) No correlation of the CRP concentration in plasma of (G) septic shock patients and of (H) reclassified patients with high SOFA score was observed with survival. Groups were compared using the non-parametric Mann-Whitney U test (∗*p* < 0.05, ∗∗*p* < 0.01, ns, not significant). Shown are means ± SEM, *n* = 7 (yes), 6 (no) in (A and E), 6 (yes), 7 (no) in (B and F), 10 (survived), 3 (deceased) in (C and G), 8 (survived), 5 (deceased) in (D and H).

To further evaluate the importance of the observed shift of S1P association from HSA to HDL in patients with surgical trauma, sepsis, and septic shock, we analyzed the amount of both S1P carrier molecules in all plasma samples. Both HSA and HDL concentrations were significantly reduced in patients compared to controls, with lowest amounts of both present in plasma of septic shock patients (Figures 5A and 5B). While HSA dropped from initially 43 g/L to 25 g/L in surgical trauma patients with low SOFA score, the concentration of HSA continued to fall only slightly with increasing SOFA score in sepsis (22 g/L) and septic shock patients (17 g/L) (Figure 5A). On the other hand, surgical trauma patients with low SOFA score showed no significant decrease of HDL. Only patients with sepsis and septic shock revealed significant reductions of HDL in plasma from initially 61 mg/dL in healthy controls to 34 mg/dL in sepsis and 13 mg/dL in septic shock patients (Figure 5B). LC-MS/MS analyses of fractionated plasma after lipoprotein precipitations demonstrated a similar loss of S1P associated with HSA in all patients compared to healthy controls (Figure 5C), while S1P bound to HDL increased significantly in all patients compared to healthy controls with surgical trauma patients showing the highest increase (Figure 5D). These patterns clearly resemble the FIDA results (Figures 2 and 3) with the important difference that plasma fractionation with LC-MS/MS analyses requires many hours of manual work, while the applied FIDA methods only take 7 min per run and can be fully automated. However, despite a clear correlation of LC-MS/MS and FIDA data, we did not find an overlap. Most interestingly, the complex sizes of S1P-FITC/HDL that resulted from the complex dissociation method were heterogeneously distributed in septic shock patients and patients with high SOFA score. We therefore correlated the resultant R_h_ with available clinical data of the septic shock patients and patients with a SOFA score greater than 8 and found a significant negative correlation with the need for mechanical ventilation (Figures 6A and 6B) and a positive correlation with the survival rate (Figures 6C and 6D). Notably, the analyzed plasma samples from patients were taken upon intensive care unit (ICU) admission. While levels of C-reactive protein (CRP) as an established sepsis marker were similarly able to predict the need for mechanical ventilation in patients with septic shock and high SOFA score (Figures 6E and 6F), CRP levels were not able to discriminate survivors from non-survivors (Figures 6G and 6H). Thus, FIDA analysis was able to clearly detect a skewed S1P-FITC binding to HDL, which turned out to be a promising marker for the prediction of sepsis severity and outcome on the day of admission to the ICU. Further studies are required to confirm the predictive value of this method and its suitability for routine analyses in the clinical setting.

## Discussion

S1P promotes endothelial cell (EC) barrier formation and lymphocyte circulation, and it has diverse functions in inflammatory processes.^22^^,^^23^^,^^24^^,^^25^^,^^26^^,^^27^ Collapse of the EC barrier is an important severity factor for the development of septic shock.^28^ Potential treatment options to promote EC barrier stabilization are highly desirable. S1P is a promising drug candidate because of its profound barrier-stabilizing activity.^22^^,^^24^ But the use of different carrier molecules has an influence on its signaling capabilities.^11^^,^^12^^,^^16^^,^^25^ The observed shift of S1P binding from HSA to HDL in patients with systemic inflammation may therefore have functional consequences. For example, low HDL-bound S1P levels correlate with coronary artery disease, which can be rescued by additional loading of HDL particles with S1P.^13^^,^^29^ In contrast, HDL-bound S1P has anti-inflammatory properties and protects the EC barrier.^16^ While most patients showed increased binding of S1P to HDL, a subgroup of septic shock patients and patients with high SOFA score revealed very low complex formation of S1P-FITC with HDL. These patients were mainly non-survivors. HDL itself correlates with the survival rate of sepsis patients, and low S1P levels in plasma are predictive of septic shock in a more sensitive and specific way than CRP.^21^ FIDA measurements revealed that very low complex formation of S1P-FITC with HDL on the day of admission to the ICU is a significant indicator of an unfavorable outcome and a much better predictor than the commonly used biomarker CRP. In addition, increased S1P-HDL complex formation due to therapeutic intervention may help to identify the success of future treatment options at a very early stage. Such a theragnostic approach could involve novel carriers of S1P like the ApoM-Fc fusion protein that already demonstrated beneficial effects in a murine sepsis model.^30^^,^^31^

FIDA is a technique mainly used to describe complex formation of binding partners by monitoring the increase of the molecular size, specifically of the R_h_. Typical applications include the characterization of antibody binding, protein stability, and intermolecular interactions.^2^^,^^3^^,^^4^^,^^5^^,^^6^^,^^7^ Our study extends the application to the identification of different carriers for S1P in human plasma samples. By applying two different methods, we separately identified complex formation of S1P-FITC with either HSA or HDL. A shift of S1P carriers in septic patients was already identified by lipoprotein precipitation followed by LC-MS/MS,^17^ and the data of both methods correlated significantly. But in contrast to LC-MS/MS, plasma can be directly used for FIDA measurements, which require no more than 7 min per analysis. In addition, FIDA identified a subgroup of septic shock patients and patients with high SOFA score that revealed very low complex formation of S1P-FITC with HDL and mainly consisted of non-survivors. This finding is specific for FIDA and was not found by LC-MS/MS, probably due to the higher variation of S1P measurements in processed samples.^17^

The applied FIDA methods allowed for a relative determination of S1P-FITC binding to HSA and HDL. The measured R_h_ was likely influenced by many different factors in plasma including the concentrations of HSA and HDL, potential modifications of these carriers like oxidation and posttranslational modifications that might affect S1P-FITC binding, and the presence of endogenous S1P. While FIDA is not able to distinguish between different influencing factors, the key advantage of this method is the possibility to trace the S1P-FITC binding behavior under native conditions regardless of the many different factors involved. The determined K_D_ for S1P-FITC binding to HSA was 20 μg/mL or 300 nM. Reported values for S1P binding are 1.3 μM^32^ and 22 μM.^33^ The stronger binding determined with FIDA could be a result of the additional FITC label of the S1P molecule, which may result in stronger binding capabilities. But it could also be a result of the employed binding assay. The reported assays were competitive binding experiments, while no competing molecules were present in the herein reported FIDA experiments. Similar reasons may apply to the determined K_D_ of 1.25 mg/mL protein for S1P-FITC binding to HDL. Considering a typical ApoM content of 30 mg/g HDL as the specific binding protein in HDL particles, the K_D_ for S1P-FITC binding to ApoM would be 1.8 μM, which is slightly higher than the reported K_D_ of 0.9 μM for the natural S1P binding to ApoM.^34^ Experiments using the kinetic exclusion assay resulted in a much lower K_D_ of 21 nM for S1P binding to HDL.^33^ Collectively, the presented FIDA binding studies revealed K_D_ values for S1P-FITC binding to HSA and HDL that were close to some, but not all, of the reported values. FIDA should be considered as a new technique for non-competitive binding studies, which may provide slightly different K_D_ values compared to competitive binding assays.

### Limitations of the study

This is the first study that determined the relative binding of S1P to its carrier molecules HSA and HDL directly in plasma samples of healthy controls and patients suffering from surgical trauma, sepsis, and septic shock using FIDA. The presented results are purely correlative and do not provide any functional relevance of the observed shift of S1P carriers in all patients compared to controls. The presented study included only a limited number of patients and healthy volunteers and needs to be confirmed with additional larger patient cohorts, which is currently in progress. Although FIDA measurements correlated significantly with endogenous S1P levels associated with HSA and HDL, the use of S1P-FITC as indicator may not exactly mirror the binding behavior of endogenous S1P. Finally, although low amounts of HDL-S1P complexes in patients with septic shock and high SOFA score significantly predicted low survival of patients on the day of admission to the ICU, the corresponding number of non-survivors in this study was too low to be considered as a representative outcome and requires further confirmation. Despite all these valid limitations, FIDA holds promise to improve patient stratification on the ICU at an early time point.

## Resource availability

### Lead contact

Further information and requests for resources and reagents should be directed to and will be fulfilled by the lead contact, Markus Gräler (Markus.Graeler@med.uni-jena.de).

### Materials availability

This study did not generate new unique reagents.

### Data and code availability

• All data reported in this paper will be shared by the lead contact upon request.
• This paper does not report original code.
• Any additional information required to reanalyze the data reported in this paper is available from the lead contact upon request.

## Acknowledgments

We thank Mareike Schilder for excellent technical assistance. Parts of this work were funded by 10.13039/501100016387Grifols SA (Barcelona, Spain) and the 10.13039/501100004403Thüringer Aufbaubank (Erfurt, Germany), co-financed by the 10.13039/501100000780European Union, project number 2022 FGI 0009.

## Author contributions

M.H.G.: writing – review and editing, writing – original draft, visualization, supervision, resources, project administration, funding acquisition, and conceptualization. I.S.: writing – review and editing, writing – original draft, visualization, methodology, investigation, formal analysis, and conceptualization. A.Z.: writing – review and editing, methodology, and investigation. A.Q.: writing – review and editing, methodology, and investigation. M.S.W.: writing – review and editing and resources. A.N.: writing – review and editing and resources. S.K.: writing – review and editing and resources. B.L.: writing – review and editing and resources.

## Declaration of interests

The authors declare no competing interests.

## STAR★Methods

### Key resources table

**Table:** 

REAGENT or RESOURCE	SOURCE	IDENTIFIER
**Chemicals, peptides, and recombinant proteins**		

S1P Fluorescein	Echelon Biosciences	S-200F
S1P	Avanti Polar Lipids	860492P
C17-S1P	Avanti Polar Lipids	860461P
Plasbumin-20	Grifols	PZN 05559806
60 × 2 MultoHigh RP18-3	CS Chromatographie Service	556201–1174

**Software and algorithms**		

Graphpad Prism	Graphpad Software	Prism 8
FIDA Analysis Software	FIDA Biosystems	FIDA Software V2.32
Analyst MS software	AB Sciex	Analyst 1.6.3

**Other**		

FIDA Analysis System	FIDA Biosystems	FIDA-1
Mass spectrometer	AB Sciex	QTrap

### Experimental model and study participant details

#### Plasma samples

For this study, caucasian patients from Western Europe who were admitted to the intensive care units (ICU) of the University Medical Center Hamburg-Eppendorf (Hamburg, Germany) with sepsis or after surgery were enrolled. Male and female patients were included with no significant sex differences between groups (Table 1). Informed consent was obtained from patients or their legal representatives. The study protocol was approved by the local Research Ethics Committee (Hamburg Chamber of Physicians: reference PV4550). EDTA plasma was obtained by centrifugation from blood samples from healthy controls (*n* = 14) and patients (*n* = 63), and immediately frozen and stored at −80°C until use. Patients were classified into the following 3 groups: (1) Patients admitted to the ICU post-surgery without infections (“surgical trauma”, *n* = 11) fulfilling at least two of the following severe inflammatory response syndrome (SIRS) criteria: temperature of more than 38°C or less than 36°C, heart rate of more than 90 beats per minute, respiratory rate of more than 20 per minute or partial pressure of arterial carbon dioxide (PaCO_2_) of less than 32 mm Hg, and white blood cell (WBC) count of more than 12 × 10^9^/L or less than 4 × 10^9^/L. (2) Patients admitted to the ICU with at least two SIRS criteria as mentioned above together with a diagnosed infection or a clinical syndrome pathognomonic for an infection (“sepsis”, *n* = 36). (3) Patients (“septic shock”, *n* = 16) admitted to the ICU with at least two SIRS criteria as mentioned above together with a diagnosed infection or a clinical syndrome pathognomonic for an infection, additional hypotension requiring vasopressor therapy to maintain a mean blood pressure of 65 mm Hg or greater plus serum lactate concentration greater than 2 mmol/L in spite of adequate fluid resuscitation and the presence of the following organ failure: acute encephalopathy, thrombocytopenia (reduction of at least 30% in 24 h or platelets of not more than 100 × 10^9^/L), hypoxia (partial pressure arterial oxygen (PaO_2_) of not more than 4.3 kPa/75 mm Hg with room air or PaO_2_/fraction of inspired oxygen (FiO_2_) of not more than 33 kPa/250 mm Hg under oxygen), renal dysfunction (urine output of not more than 0.5 mL/kg per h in 2 h despite sufficient fluids), resuscitation or increase of serum creatinine (of more than 2-fold) or both, metabolic acidosis (base excess of not more than −5 mmol/L or lactate of more than 1.5 mmol/L), or hypotension/cardiovascular dysfunction (after fluid resuscitation with at least 30 mL/kg of crystalloid).^17^^,^^21^ Patients were also categorized according to the sequential organ failure assessment (SOFA) score, which was generated for each patient (Table 1). Patients were included on day 1 after being diagnosed with sepsis, or after surgery, and plasma was taken within 24 h after inclusion. Patients with HSA used for fluid resuscitation, with an age <18 years, pregnancy, or a moribund status were excluded. Healthy volunteers were used as controls. The Institute of Clinical Chemistry and Laboratory Medicine at the University Medical Center Hamburg-Eppendorf (Hamburg, Germany) measured the concentration of HDL and HSA together with other markers including C-reactive protein (CRP).

### Method details

#### Flow induced dispersion analysis

Plasma samples were analyzed using the FIDA 1 instrument (Fida Biosystems ApS, Copenhagen, Denmark) employing light-emitting-diode (LED) induced fluorescence detection with an excitation wavelength of 480 nm and emission wavelength > 515 nm. Flow induced dispersion analysis (FIDA) is a technology that relies on the analysis of a parabolic hydrodynamic flow profile for measuring the apparent hydrodynamic radius (R_h_) of a selective ligand (indicator) as it binds to an analyte of interest. In this study, we used FITC-labelled S1P (Echelon Biosciences) as indicator and patient plasma diluted to 10% in PBS as analyte. Three different methods can be employed for the FIDA analysis: the pre-incubation (PreMix) method, where indicator and analyte are pre-mixed (>10 min) prior to the FIDA measurements, and subsequently pushed through the capillary using the analyte solution; the capillary mix (CapMix) method, where indicator and analyte are mixed directly in the capillary during the mobilization period; and the complex dissociation (CapDis) method, where indicator and analyte are pre-incubated (>10 min) prior to the FIDA measurements, and then mobilized through the capillary using PBS.^1^ In this study, we used the CapMix and CapDis methods with the following protocol: first, the uncoated capillary was rinsed with 1 M NaOH (pressure 3500 mbar, time 45 s), followed by a capillary equilibration step with PBS (3500 mbar for 75 s). Subsequently, the analyte solution was filled into the capillary (3500 mbar for 30s) followed by the indicator sample (50 mbar for 10 s). Finally, the indicator sample was mobilized to the fluorescence detector with analyte solution (400 mbar for 200 s). For binding curves and competition experiments, Plasbumin-20 HSA (Grifols Deutschland GmbH, Frankfurt, Germany) was used.

#### Isolation of high-density lipoprotein

High-density lipoprotein (HDL) was isolated from plasma of healthy human volunteers by sequential density gradient ultracentrifugation.^29^ The density of the plasma samples was increased to 1.210 g/mL by adding potassium bromide (KBr, 0.325 g/mL plasma). The density gradient was then constructed in ultracentrifuge tubes with initial 2 mL of a sodium chloride-potassium bromide (NaCl-KBr) solution of density (d) 1.240 g/mL at the bottom of the tube. The following solutions were then layered on top of this solution: Three mL of plasma at d 1.21 g/mL, 2 mL of NaCl-KBr solution of d 1.063 g/mL, 2.5 mL of d 1.019 g/mL, and 3 mL of NaCl solution of d 1.006 g/mL at ambient temperature. The gradient was centrifuged at 274,000 rcf for 48 h. The resulting band III (from the top) of about 1.3 mL was determined as HDL_2_, band IV (about 1.6 mL) and the intermediate fraction below band IV of about 0.5 mL corresponded to HDL_3_ and were pooled for the analysis. Protein concentration of isolated HDL was determined by Bradford assay (Bio-Rad, Feldkirchen, Germany).

#### Lipoprotein precipitation

Lipoproteins were sequentially precipitated via an increasing Na_3_P(W_3_O_10_)_4_ concentration.^17^ Chylomicrons and very low-density lipoproteins (VLDL) were precipitated by adding 25 μL of 1% Na_3_P(W_3_O_10_)_4_ and 25 μL of a 2 M MgCl_2_ solution to 500 μL plasma. The solution was mixed and incubated for 15 min at ambient temperature. After centrifugation at ambient temperature for 10 min at 6000 rcf, the precipitated VLDL fraction was isolated and the supernatant was transferred to new tubes. Low-density lipoproteins (LDL) were subsequently precipitated by addition of 25 μL of 4% Na_3_P(W_3_O_10_)_4_ solution to the first supernatant. After incubation for 15 min at ambient temperature, samples were centrifuged for 10 min at 6,000 rcf. The supernatant was separated from the precipitated LDL and transferred into a new tube. HDL were precipitated by adding 25 μL of a 40% Na_3_P(W_3_O_10_)_4_ solution to the second supernatant. Samples were incubated for 2 h at ambient temperature and centrifuged for 30 min at 20,000 rcf. The resultant supernatant was the lipoprotein-free HSA-containing fraction.

#### Extraction and quantification of S1P

S1P measurements were performed according to an established protocol using liquid chromatography-tandem mass spectrometry (LC-MS/MS).^17^ Samples were adjusted to a final volume of 1 mL with H_2_O after addition of C17-S1P as internal standard (100 pmol/sample, Avanti Polar Lipids, Alabaster, AL, USA). After addition of 0.3 mL 6 N HCl, 1 mL methanol and 2 mL chloroform in glass centrifuge tubes, samples were vigorously vortexed for 10 min. After centrifugation at 4°C for 3 min at 1,900 rcf, the lower chloroform phase was transferred into a new glass centrifuge tube. An additional amount of 2 mL chloroform was added to the aqueous solution, and the extraction procedure was repeated. The two chloroform phases were combined and vacuum-dried at 60°C for 50 min using a vacuum concentrator. The extracted lipids were dissolved in 100 μL methanol/chloroform (4:1, v/v). Quantification of S1P was performed with the QTrap triple-quadrupole mass spectrometer (AB Sciex, Darmstadt, Germany) interfaced with the 1100 series chromatograph (Agilent, Waldbronn, Germany) using positive electrospray ionization (ESI). Multiple reaction monitoring (MRM) transitions were 380/264 for S1P and 366/250 for the internal standard C17-S1P. A 2 mm × 60 mm MultoHigh C18 reversed phase column with 3 μm particle size was used for chromatographic separation of all analytes (CS Chromatographie Service GmbH, Langerwehe, Germany). The column was equilibrated with 10% methanol and 90% of 1% formic acid in H_2_O for 5 min, followed by 10 μL sample injection and 15 min elution with 100% methanol with a flow rate of 300 μL/min. Standard curves were generated by adding increasing concentrations of S1P to 100 pmol of the internal standard C17-S1P. Linearity of the standard curves and correlation coefficients were obtained by linear regression analyses. Data analyses were performed using Analyst 1.6.3 (AB Sciex).

### Quantification and statistical analysis

#### Statistical analysis

Statistical analysis and graphical representation of the data were performed using the GraphPad Prism 8 software. P-values were calculated using Student’s T-test, non-parametric Mann-Whitney U test, or Spearman correlation test as specified in the figure legends. The study cohort is described in Table 1. *p* < 0.05 was considered statistically significant.
